# ANP32E induces tumorigenesis of triple‐negative breast cancer cells by upregulating E2F1

**DOI:** 10.1002/1878-0261.12202

**Published:** 2018-04-18

**Authors:** Zhenchong Xiong, Liping Ye, He Zhenyu, Fengyan Li, Yahui Xiong, Chuyong Lin, Xianqiu Wu, Guangzheng Deng, Wei Shi, Libing Song, Zhongyu Yuan, Xi Wang

**Affiliations:** ^1^ Department of Breast Surgery State Key Laboratory of Oncology in Southern China Collaborative Innovation Center for Cancer Medicine Sun Yat‐sen University Cancer Center Guangzhou China; ^2^ Department of Experimental Research State Key Laboratory of Oncology in Southern China Collaborative Innovation Center for Cancer Medicine Sun Yat‐sen University Cancer Center Guangzhou China; ^3^ Department of Radiation Oncology State Key Laboratory of Oncology in Southern China Collaborative Innovation Center for Cancer Medicine Sun Yat‐sen University Cancer Center Guangzhou China; ^4^ The First College of Clinical Medicine Southern Medical University Guangzhou China; ^5^ Department of Medical Oncology State Key Laboratory of Oncology in Southern China Collaborative Innovation Center for Cancer Medicine Sun Yat‐sen University Cancer Center Guangzhou China

**Keywords:** ANP32E, cell cycle, E2F1, TNBC, tumorigenesis

## Abstract

Triple‐negative breast cancer (TNBC) lacks expression of estrogen receptor (ER), progesterone receptor, and the HER2 receptor; it is highly proliferative and becomes the deadliest forms of breast cancer. Effective prognostic methods and therapeutic targets for TNBC are required to improve patient outcomes. Here, we report that acidic nuclear phosphoprotein 32 family member E (ANP32E), which promotes cell proliferation in mammalian development, is highly expressed in TNBC cells compared to other types of breast cancer. High expression of ANP32E correlates significantly with worse overall survival (OS; *P *<* *0.001) and higher risks of disease recurrence (*P *<* *0.001) in patients with TNBC. Univariate and multivariate Cox‐regression models show that ANP32E is an independent prognostic factor in TNBC. Furthermore, we discovered that ANP32E promotes tumor proliferation *in vitro* by inducing G1/S transition, and ANP32E inhibition suppresses tumor formation *in vivo*. By examining the expression of E2F1, cyclin E1, and cyclin E2, we discovered that ANP32E promotes the G1/S transition by transcriptionally inducing E2F1. Taken together, our study shows that ANP32E is an efficient prognostic marker, and it promotes the G1/S transition and induces tumorigenesis of TNBC cells by transcriptionally inducing E2F1.

**Abbreviations**
ANP32E: acidic nuclear phosphoprotein 32 family member E
CI: confidence interval
DMFS: distant metastasis‐free survival
ER: estrogen receptor
H&E: hematoxylin/eosin
HRs: hazard ratios
IHC: immunohistochemistry
OS: overall survival
PR: progesterone receptor
qRT–PCR: real‐time quantitative PCR
RFS: relapse‐free survival
TCGA: The Cancer Genome Atlas
TNBC: triple‐negative breast cancer

## 1 Introduction

Triple‐negative breast cancer (TNBC) is a subtype of breast cancer that is highly proliferative and has a poor prognosis (Li *et al*., 2018). Patients with TNBC have been reported to have a higher risk for disease relapse within the first 3–5 years of diagnosis (Dietze *et al*., 2015; Foulkes *et al*., 2010; Qiu *et al*., 2016). However, because of the negative expression for the estrogen receptor (ER), progesterone receptor (PR), and HER2 receptor, the prognostic markers and therapeutic targets of TNBC remain largely unknown (Cianfrocca and Gradishar, 2009; Denkert *et al*., 2016; Foulkes *et al*., 2010). Moreover, studies also suggested that the pathological variables (including tumor mass and lymph node involvement) are not efficient prognostic factors for patients with TNBC (Dietze *et al*., 2015; Muellner *et al*., 2015; Park *et al*., 2011; Qiu *et al*., 2016; Stoeck *et al*., 2014). Prognostic markers that could efficiently predict disease relapse or distant metastasis are urgently needed.

Sustained proliferation, which is induced by aberrant regulation of the cell cycle, is suggested to be critical for TNBC tumor progression (Bi *et al*., 2015; Hanahan and Weinberg, 2011; Ossovskaya *et al*., 2011; Otto and Sicinski, 2017). The G1/S phase is the most critical rate‐limiting step in cell cycle promotion, which involves a transcriptional complex that includes Rb and E2F1 and two cell cycle kinases, CDK2–cyclin E and CDK2–cyclin A (Cam and Dynlacht, 2003; Chan and Nimnual, 2010). E2F1 is a transcription factor involved in cell cycle regulation and cell proliferation. During the cell cycle, Rb becomes phosphorylated and detaches from E2F1, which renders E2F1 transcriptionally active. E2F1 then promotes cell cycle progression by regulating cell cycle genes, such as cyclin E1, cyclin E2, and cyclin A (Duan *et al*., 2016; Taylor‐Harding *et al*., 2015; Ye *et al*., 2016). Furthermore, cyclins bind to CDK2, which in turn induces the G1/S transition (CDK2–cyclin E) and S‐phase entry (CDK2–cyclin A). Aberrant regulation of E2F1/cyclin/CDK2 promotes tumor progression in breast cancer. Several studies have shown that the expression of cell cycle‐related genes significantly correlated with poor outcomes in patients with breast cancer (Hunt *et al*., 2016; Keyomarsi *et al*., 2002; Magbanua *et al*., 2015; Ye *et al*., 2016). For instance, by detecting the expression of cytoplasmic cyclin E, Kelly K. Hunt suggested that cyclin E could identify patients with the highest likelihood of recurrence in breast cancer (Hunt *et al*., 2016). Low expression of E2F1 significantly correlates with favorable breast cancer outcomes (Vuaroqueaux *et al*., 2007). Thus, studies of the molecular mechanisms of cell cycle progression are needed to discover novel prognostic factors and therapeutic targets.

Acidic nuclear phosphoprotein 32 family member E (ANP32E) belongs to a family of proteins with leucine‐rich repeats, which involve a large number of biological functions, such as cell adhesion, early mammalian development, and cancer metastasis (Kobe and Kajava, 2001; Li *et al*., 2017; Matilla and Radrizzani, 2005; Radrizzani *et al*., 2001; de Wit *et al*., 2011). ANP32E is a specific H2A.Z histone chaperone that removes H2A.Z from enhancer and insulator regions of a target gene and regulates its expression (Farris *et al*., 2005; Gursoy‐Yuzugullu *et al*., 2015; Mao *et al*., 2014; Obri *et al*., 2014). Studies also reported that ANP32E is involved in cerebellar development and synaptogenesis (Jiang *et al*., 2002; Matilla and Radrizzani, 2005; Radrizzani *et al*., 2001). In breast cancer, a six‐gene signature consisting of *DSC2, TFCP2L1, UGT8, ITGB8, ANP32E*, and *FERMT1* is associated with lung metastasis (Landemaine *et al*., 2008).

Here, we discovered that ANP32E is highly expressed in TNBC cells compared to other types of breast cancer, and high levels of ANP32E expression are associated with shorter survival times and higher risks of disease relapse in TNBC. Furthermore, we demonstrated that ANP32E promotes G1/S progression by upregulating E2F1 expression and, consequently, contributes to the proliferation and tumorigenesis of TNBC cells. This may provide a potential prognostic tool in the treatment of TNBC.

## 2 Materials and methods

### 2.1 Cell lines and cell culture

Breast cancer cell lines (MDA‐MB‐361, BT‐474, ZR‐75‐30, SK‐BR‐3, BT‐549, MDA‐MB‐231, MDA‐MB‐468, and 4T1) were provided by the American Type Culture Collection (ATCC), and SUM159PT breast cancer cell line was provided by Asterand Bioscience. Cell lines were cultured under the following condition: 10% fetal bovine serum (HyClone, Logan, UT, USA) was added to Dulbecco's Modified Eagle Medium (Invitrogen, Carlsbad, CA, USA) under a 5% CO_2_ atmosphere at 37 °C.

### 2.2 Patients and tissue specimens

We collected 422 cancer specimens from patients with a histopathological diagnosis of breast cancer at the Sun Yat‐sen University Cancer Center from 2003 to 2012. The clinical and pathological characteristics are listed in Table 1. The timing of death or disease recurrence was determined by clinical review or a telephone interview. Fresh tumor tissues were collected from individuals diagnosed with breast cancer at the Sun Yat‐sen University Cancer Center Department of Breast Surgery. Our study was in accordance with the Declaration of Helsinki and was ethically approved by the institutional review board.

**Table 1:** Clinicopathological characteristics of patients with breast cancer

Characteristics	No. of cases (%)
Age (year)	
≤ 45	188 (44.5)
> 45	234 (55.5)
T classification	
T1–2	371 (87.9)
T3–4	37 (8.8)
N classification	
N0	203 (48.1)
N1–2	166 (39.3)
N3	53 (12.6)
ER	
Positive	129 (30.6)
Negative	293 (69.4)
PR	
Positive	125 (29.6)
Negative	297 (70.4)
Her‐2 receptor	
Amplification	59 (14.0)
Nonamplification	344 (81.5)
2+	19 (4.5)
Triple‐negative breast cancer	
Yes	238 (56.4)
No	184 (43.6)
Vital status (at follow‐up)	
Alive	324 (76.8)
Dead	98 (23.2)
Relapse status	
Relapse	110 (26.1)
Relapse‐free	312 (73.9)
Menopause status	
Premenopausal	238 (56.4)
Menopausal	184 (43.6)
Expression of ANP32E	
Low expression	240 (56.9)
High expression	182 (43.1)

### 2.3 Real‐time quantitative PCR (qRT–PCR)

Materials for real‐time quantitative PCR (qRT–PCR) were as follows: RNA from cell line and breast cancer tissue were extracted with TRIzol reagent (Invitrogen). Added with RNase‐free DNase, 2 μg RNA per sample was used for cDNA synthesis. Real‐time PCR was used to assess the mRNA level of indicated gene in cell lines and tumor tissues. The cDNA were augmented and assessed with dye SYBR Green I (Molecular Probes, Eugene, OR, USA) and ABI Prism 7500 Sequence Detection System (Applied Biosystems, Logan, UT, USA). The specific sequence of primers is listed in the Table S4.

### 2.4 Western blotting

Western blotting was performed according to previously published methods (Li *et al*., 2008). Briefly, after removal of the culture medium, cells were incubated with lysis buffer (Sigma, Saint Louis, MO, USA). The protein concentration was assessed using the bicinchoninic acid assay (Pierce, Rockford, IL, USA) according to the manufacturer's instructions. An ANP32E polyclonal rabbit antibody (1 : 800 dilution; Abnova Corporation, Taipei City, Taiwan), anti‐E2F1 monoclonal mouse antibody (1 : 800 dilution; Cell signaling Technology, Danvers, MA, USA), anticyclin E1 monoclonal mouse antibody (1 : 800 dilution; Proteintech, Chicago, IL, USA), anticyclin E2 monoclonal mouse antibody (1 : 800 dilution; Proteintech), anti‐Rb monoclonal mouse antibody (1 : 2000 dilution; Cell Signaling Technology), anti‐p‐Rb rabbit polyclonal antibody (1 : 1000 dilution; Cell Signaling Technology), anti‐α‐tubulin monoclonal mouse antibody (1 : 3000 dilution; Sigma), and goat anti‐mouse immunoglobulin G secondary antibody (1 : 1500 dilution; Pierce) were used. Fold changes of the protein level were evaluated with imagej software (https://imagej.nih.gov/ij/). The first stripe in each roll of protein sample was defined as reference substance. The fold change of each sample was based on the gray values as follow: the gray value of the targeted stripe/the gray value of the reference stripe.

### 2.5 Immunohistochemistry

Immunohistochemistry (IHC) was performed to assess protein expression in 422 breast cancer tissues as previously described (Lin *et al*., 2011). The immunostaining intensity of tissues was reviewed and quantified separately by two pathologists. Scores were obtained by combining the proportion of positively staining cells and the staining intensity. Staining intensity was classified as follows: 1, no staining; 2, weak staining (light yellow); 3, moderate staining (yellow brown); 4, strong staining (brown). The proportion of positively staining cells was recorded according to the following criteria: 0, no positive cells; 1, < 10% positive cells; 2, 10%–35% positive cells; 3, 35%–75% positive cells; and 4, > 75% positive cells. Expression levels of the indicated proteins were determined using the staining index (SI), which was scored as the product of the proportion of positive‐staining cells and the staining intensity score. The SI consisted of possible scores of 0, 2, 3, 4, 6, 8, 9, 12, and 16. High expression and low expression of protein were defined as SI ≥ 8 and SI < 8, respectively. A measure of heterogeneity based upon the log‐rank test with regard to overall survival (OS) was performed to determine cutoff values.

The immunostaining intensity of each tested specimen was determined by the method of mean optical density (MOD), which was previously demonstrated (Lin *et al*., 2015). The stained sections were reviewed at 200× magnification, and MOD, which was identified using 10 randomly selected fields in each section, represented the strength of signal as measured per positive pixels.

### 2.6 Plasmids, virus constructs, and retroviral infection of target cells

The pGL3 luciferase reporter plasmid (Promega, Madison, WI, USA) was used to construct luciferase reporter plasmids of E2F1. The Lipofectamine 3000 reagent (Invitrogen) was used for transfection of the luciferase reporter plasmid according to the manufacturer's instruction. After amplification, human ANP32E or E2F1 cDNA was cloned into the pSin‐puro‐retro vector (Clontech, Mountain View, CA, USA). Short‐hairpin RNA (shRNA) targeting ANP32E or E2F1 in a vector with pLKO‐puro were used (Sigma‐Aldrich). Next, 2 × 10^4^ cells were added and infected by a retrovirus that was produced by pLKO‐puro‐ANP32E‐shRNA transfection into 293FT cells for 72 h. Subsequently, these cells were transfected with the pMSCV‐neo‐luci plasmid. Cells expressing ANP32E‐shRNA‐luci were cultured with 250 μg·mL^−1^ G418 and 0.5 μg·mL^−1^ puromycin for 10 days to produce stable cell lines.

### 2.7 Colony formation assay

Cells (5 × 10^2^ per plate) were cultured on 6‐well plates for 14 days. Colonies formed by the cells were treated as previously reported (Lin *et al*., 2010).

### 2.8 Anchorage‐independent growth ability assay

After trypsinization, cells were suspended in 0.3% agar (Sigma) plus 2 mL of complete medium in a 6‐well plate (5 × 10^3^ cells per well). Cells mixed with culture medium were plated above a layer containing a mixture of 0.66% agar plus medium. Colonies were counted after 10 days.

### 2.9 Flow cytometry analysis

Before analysis on a flow cytometer (FACSCalibur; BD Biosciences, San Jose, CA, USA), 2 × 10^4^ cells were washed, fixed, pelleted, and incubated in bovine pancreatic RNAse (Sigma). They were then stained with propidium iodide (Sigma‐Aldrich). The specific procedure was previously reported (Lin *et al*., 2010).

### 2.10 Xenograft tumor model, IHC, and hematoxylin/eosin (H&E) staining

Female BALB/c‐nude mice (4–5 weeks old, 18–20 g) were provided by the Hunan SJA Laboratory Animal Co, Ltd. in Hunan, China. All BALB/c‐nude mice were inoculated *in situ* with 4T1‐Vector‐luci cells (1 × 10^5^)/SUM‐159PT‐Vector‐luci cells (1 × 10^6^) in the right breast and 4T1‐ANP32E‐RNAi#1 cells (1 × 10^5^)/SUM‐159PT‐ ANP32E‐RNAi#1 cells (1 × 10^6^) in the left breast. Tumors were measured every 3 days beginning 7 days after inoculation, and all the mice were sacrificed at 28 days after inoculation. The tumors were paraffin‐embedded and stained for IHC using an anti‐Ki‐67 mouse antibody (1 : 100 dilution; Cell Signaling Technology) and hematoxylin/eosin (H&E). Expression of Ki‐67 was calculated by the percentage of ki‐67‐positive cells: High expression and low expression of protein were defined as ≥ 14% and < 14%, respectively (Cheang *et al*., 2009). All experimental procedures were approved by the Institutional Animal Care and Use Committee of Sun Yat‐sen University.

### 2.11 Luciferase activity assay

A total of 3 × 10^3^ cells were cultured in 48‐well plates for 1 day. The control luciferase plasmid or luciferase reporter plasmids (100 ng) were added to 1 ng pRL‐TK Renilla plasmid (Promega) and transfected into cells using the Lipofectamine 3000 reagent (Invitrogen). After 24 h following transfection, luciferase and Renilla signals were measured using the Dual‐Luciferase Reporter Assay Kit (Promega) according to the manufacturer's protocol.

### 2.12 Statistical analysis

Statistical analyses were performed with the spss 20.0 (IBM, Chicago, IL, USA) statistical software package. The clinicopathological characteristics of groups of patients defined by ANP32E expression were analyzed using the chi‐square test. The Kaplan–Meier method and log‐rank test were used for survival analysis. Univariate and multivariate Cox‐regression models were used to assess the survival data. *P *<* *0.05 was considered statistically significant.

A subgroup survival analysis was performed by following methods previously reported (Badwe *et al*., 2015). Patients were prestratified as factors that included TNBC, HR‐negative expression (hormone receptor), HER2‐negative expression, age (≤ 45 and > 45), and menopause status (premenopause versus menopause). Cox proportional hazards models were used to assess the mortality risk [hazard ratio (HR) and 95% confidence interval (CI)] in each subgroup. The results were displayed in a forest plot.

## 3 Results

### 3.1 ANP32E is significantly upregulated in TNBC cells

**Figure 1:**
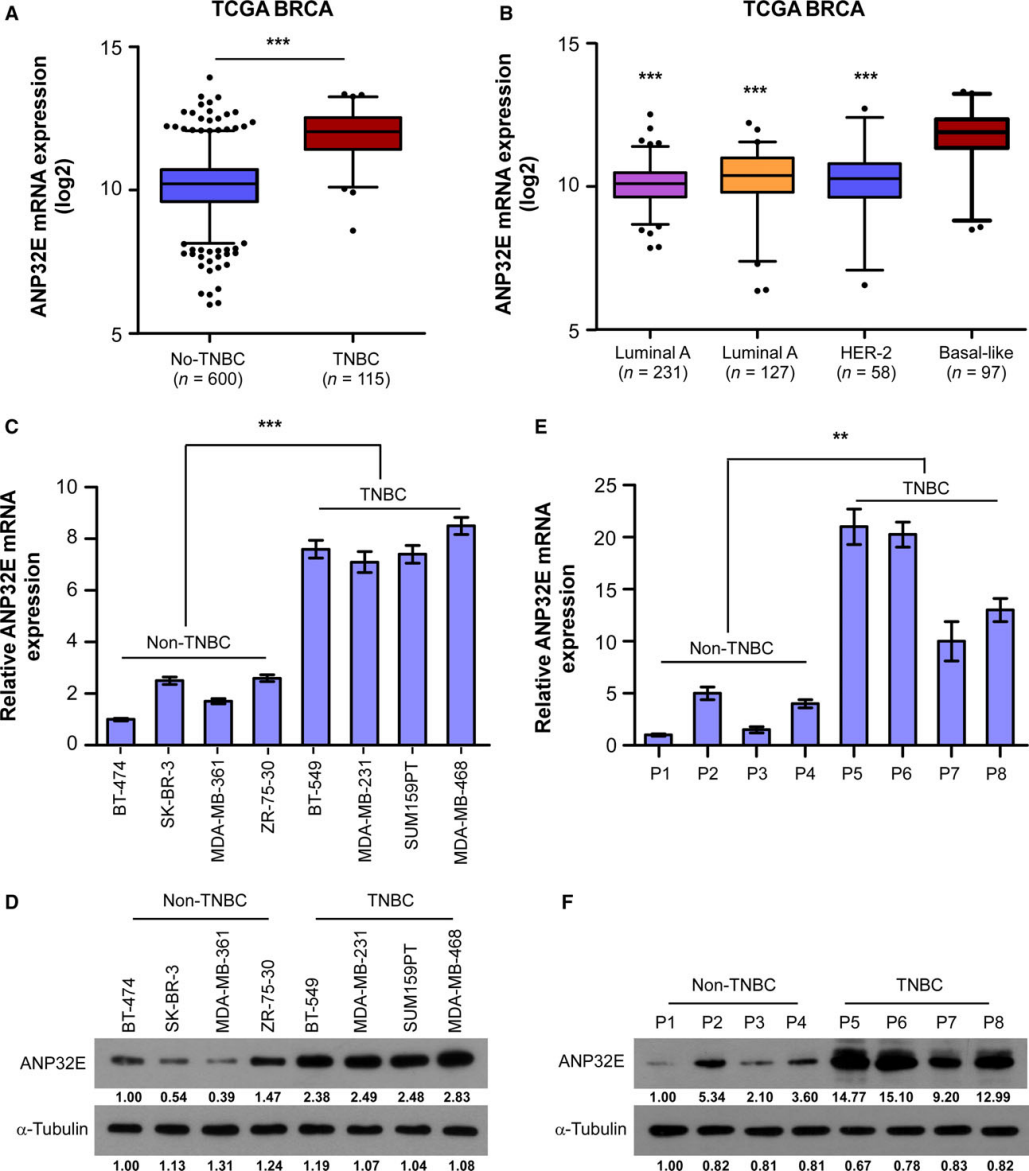
ANP32E is upregulated in TNBC cells. (A,B) *ANP32E* mRNA levels in breast cancer tissues were assessed by analyzing a TCGA breast cancer mRNA dataset of (A) non‐TNBC (*n* = 600) versus TNBC tissues (*n* = 115) and (B) molecular subtypes of breast cancer including luminal A (*n* = 231), luminal B (*n* = 127), HER2 (*n* = 58), and basal‐like (*n* = 97). Data were obtained from the TCGA data portal. (C, D) The mRNA (C) and protein (D) expression levels of ANP32E in breast cancer cell lines. (E,F) Detection of mRNA (E) and protein (F) expression levels of ANP32E in non‐TNBC and TNBC tissues. The mRNA expression and protein expression were normalized to GAPDH and α‐tubulin, respectively. Fold changes of the protein level were evaluated by ImageJ (https://imagej.nih.gov/ij/). Data are the means ± SD of three independent experiments. **P <* *0.01, ***P <* *0.001.

To study the relationship between ANP32E and TNBC, we assessed the expression of ANP32E among different types of breast cancer using the The Cancer Genome Atlas (TCGA) database. We observed a significantly higher level of ANP32E expression in TNBC cells compared to non‐TNBC cells (*P *<* *0.001, Fig. 1A). With regard to molecular subtypes of breast cancer, ANP32E expression in basal‐like breast cancer cells, which shares similar features with TNBC, was higher than the ANP32E expression in other subtypes (luminal A, luminal B, HER‐2; *P *<* *0.001, Fig. 1B). qRT–PCR showed a significantly higher mRNA expression level for *ANP32E* in TNBC cell lines (Fig. 1C). Consistently, we observed that ANP32E protein is significantly enriched in TNBC cell lines compared to non‐TNBC cell lines by western blotting (Fig. 1D). Furthermore, we randomly chose eight breast cancer tissues (four TNBC tissues and four non‐TNBC tissues) to assess protein and mRNA expression levels of ANP32E. Intriguingly, both western blotting and qRT–PCR confirmed the higher expression levels of ANP32E in TNBC tissues compared to non‐TNBC tissues (Fig. 1E,F).

### 3.2 ANP32E upregulation is associated with poorer prognoses for TNBC patients

**Figure 2:**
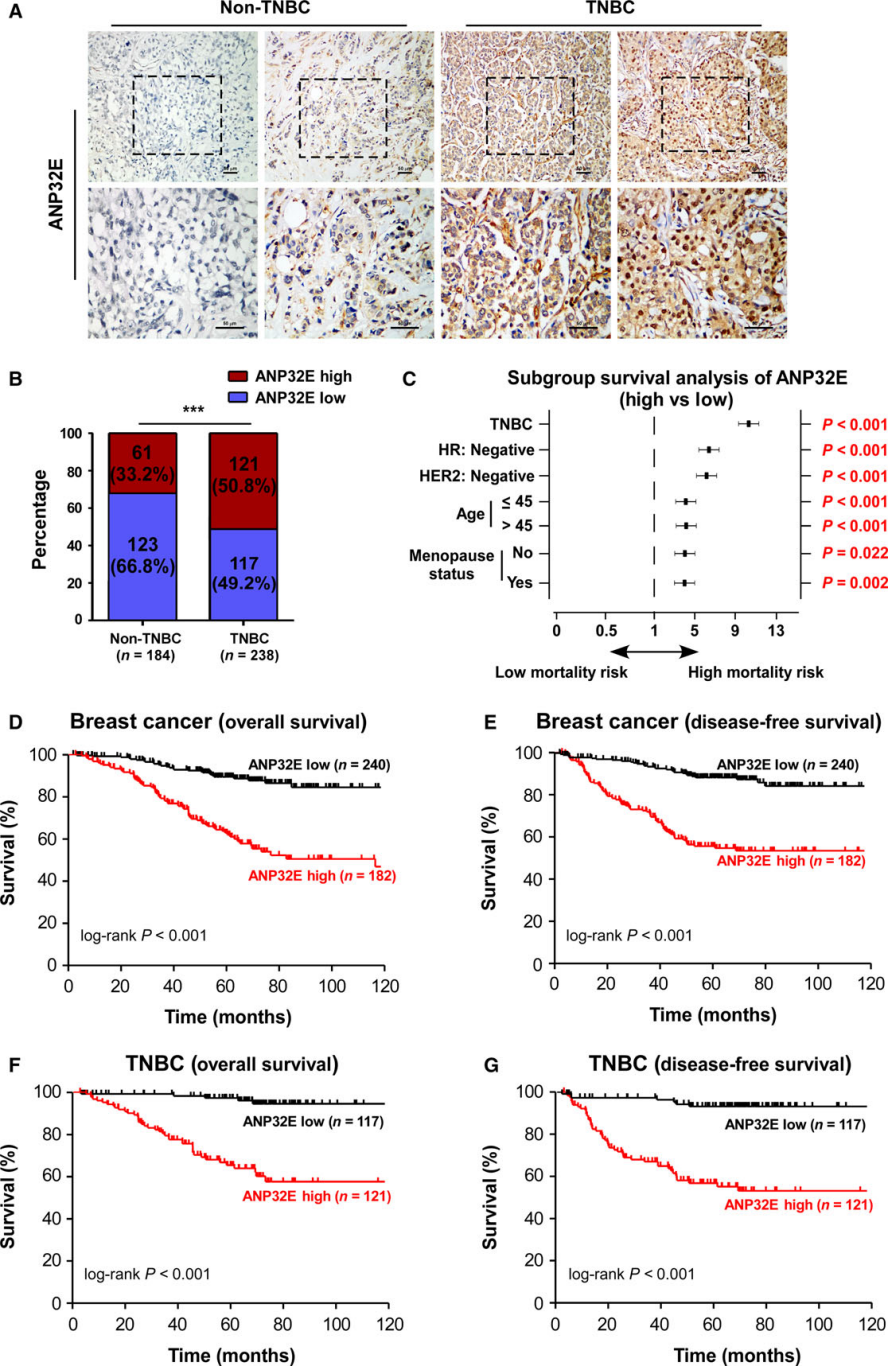
High expression of ANP32E is associated with a poor prognosis in TNBC patients. (A) Representative IHC image of ANP32E expression in TNBC and non‐TNBC specimens. (B) Statistical quantification of ANP32E staining in TNBC (*n* = 238) and non‐TNBC tissues (*n* = 184). (C) Forest plot of the OS subgroup analysis with respect to ANP32E expression: ANP32E‐high versus ANP32E‐low. High ANP32E expression significantly correlated with high mortality risk in subgroups of patients with breast cancer, including TNBC, HR‐negative, HER2‐negative, age (≤ 45 and > 45), and menopause status (premenopause and menopause). (D, E) Kaplan–Meier curve of OS (D) and DFS (E) for breast cancer patients with low expression of ANP32E (ANP32E‐low; *n* = 240) versus high expression of ANP32E (ANP32E‐high; *n* = 182; *P *<* *0.001; *P *<* *0.001; log‐rank test for OS and DFS, respectively). (F,G) Kaplan–Meier curve of OS (F) and DFS (G) for TNBC patients with low (ANP32E‐low; *n* = 117) versus high expression of ANP32E (ANP32E‐high; *n* = 121; *P *<* *0.001; *P *<* *0.001; log‐rank test for OS and DFS, respectively). DMFS, distant metastasis‐free survival; RFS, relapse‐free survival. ***P <* *0.001.

We collected 422 breast cancer tissues, including 184 non‐TNBC cases and 238 TNBC cases. First, we quantified protein expression levels of ANP32E by IHC. As shown in Table 1, 182 cases had high levels of ANP32E and 240 cases had low levels of ANP32E. ANP32E staining was weak in non‐TNBC tissues, while more intense staining was observed in TNBC tissues (Fig. 2A,B).

Next, we analyzed the correlation between ANP32E expression measured by IHC and clinicopathological characteristics of patients with breast cancer (Table S1). The chi‐squared test showed that higher expression of ANP32E was positively associated with ER‐negative samples (*P *<* *0.001) and PR‐negative samples (*P *<* *0.001; Table S1). The Kaplan–Meier survival curve showed that high expression of ANP32E correlated with shorter OS (*P *<* *0.001) and disease‐free survival (DFS; *P *<* *0.001) in patients with breast cancer (Fig. 2D,E). Among patients with TNBC, high ANP32E expression significantly correlated with shorter OS and DFS (OS, *P *<* *0.001; DFS, *P *<* *0.001; Fig. 2F,G). Univariate and multivariate analyses showed that high expression of ANP32E is an independent prognostic factor for patients with TNBC after adjusting for T stage, N stage, age, and menopause status (HR = 9.809, *P *<* *0.001; Table 2). Subgroup survival analysis showed that high expression of ANP32E correlated with higher mortality risks among patients with HR‐ or Her‐2‐negative breast cancer (HR = 6.421, *P *<* *0.001; HER2 = 6.186, *P *<* *0.001; respectively; Fig. 2C, Table S3). These results suggested that higher expression of ANP32E correlated with worse prognoses for patients with TNBC.

**Table 2:** HR for women with TNBC (univariate and multivariate)

Characteristic	TNBC			
	Univariate HRs (95% CI)	*P*	Multivariate HRs (95% CI)	*P*
Age (> 45 vs ≤ 45)	2.255 (1.186–4.288)	**0.013**	2.552 (1.271–5.122)	**0.008**
T classification (T3–4 vs T1–2)	4.626 (2.136–10.018)	**< 0.001**	4.516 (2.010–10.146)	**< 0.001**
N classification		**0.001**		**< 0.001**
N0	1		1	
N1–2	2.464 (1.267–4.791)	0.008	2.430 (1.169–5.052)	0.017
N3	5.049 (2.183–11.677)	**< 0.001**	5.737 (2.382–13.818)	< 0.001
Menopause status (Menopause vs menses)	2.105 (1.163–3.808)	**0.014**	NA	0.769
Expression of ANP32E (High vs low)	10.349 (4.079–26.253)	**< 0.001**	9.809 (3.808–25.269)	**< 0.001**

Bold indicates significant value (*P* < 0.05).

**Figure 3:**
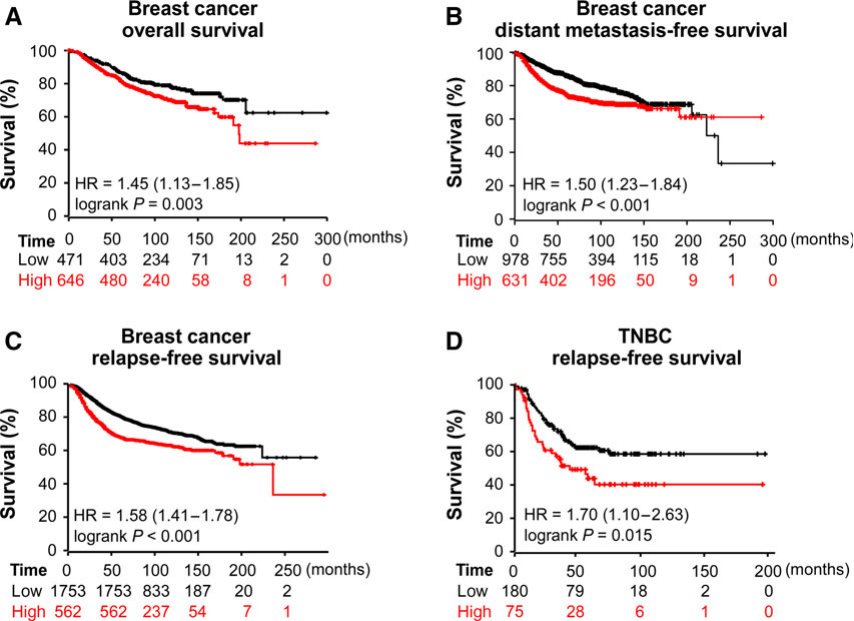
Kaplan–Meier plots of the association between ANP32E expression and outcomes in breast cancer patients. (A–C) High expression of ANP32E correlated with worse OS (A) (*n* = 1117; *P *=* *0.003), DMFS (B) (*n* = 1609; *P *<* *0.001), and RFS (C) (*n* = 2315; *P *<* *0.001) in patients with breast cancer. (D) High expression of ANP32E correlated with worse RFS (*n* = 255; *P *=* *0.015) in patients with TNBC. Data were obtained from the Kaplan–Meier plotter (http://kmplot.com/analysis/index.php?p=background). DMFS, distant metastasis‐free survival; RFS, relapse‐free survival.

Furthermore, using the Kaplan–Meier Plotter database, we observed that patients with high expression of ANP32E were correlated with shorter OS (HR = 1.45, *P *=* *0.003) and higher probability of disease relapse in breast cancer (HR = 1.58, *P *<* *0.001), and high expression of ANP32E predicted higher risk of distant metastasis in 200 months of follow‐up (HR = 1.50, *P *<* *0.001; Fig. 3A–C). Among patients with TNBC, high ANP32E expression correlated with shorter DFS (HR = 1.70, *P *=* *0.015; Fig. 3D).

### 3.3 ANP32E induces proliferation by promoting the G1/S transition in TNBC cells

**Figure 4:**
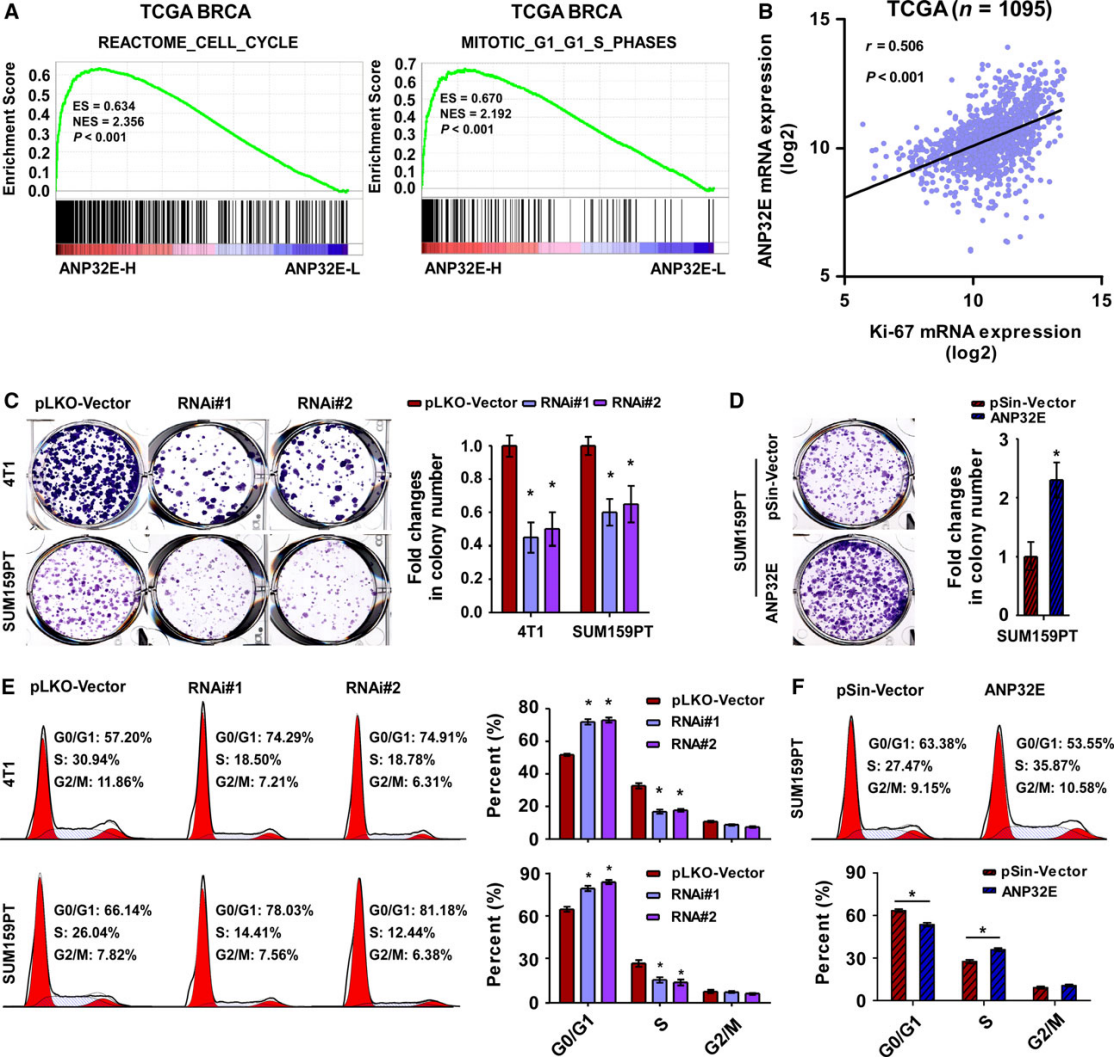
ANP32E induces cell proliferation by promoting the G1/S transition in TNBC cells. (A) The GSEA plot generated using GSEA v2.2.0 (http://www.broadinstitute.org/gsea/) showed that high ANP32E expression positively correlated with cell cycle‐related (REACTOME_CELL_CYCLE) and G1/S‐related (MITOTIC_G1_G1_S_PHASE) signatures based on the TCGA *BRCA* (TNBC) mRNA dataset. (B) Correlation between mRNA levels of *ANP32E* and *Ki‐67* based on the TCGA *BRCA* mRNA dataset. *r*, Pearson correlation coefficient; *P *<* *0.001. (C, D) Representative images (left) and quantification (right) of colony formation for the ANP32E‐inhibited (C) and ANP32E‐overexpressing (D) cell lines. (E and F) Flow cytometric analysis of indicated cell lines. *P <* *0.05.

To further explore the biological functions of ANP32E in TNBC, we performed a gene set enrichment analysis (GSEA) using mRNA expression data from the TCGA database and discovered that high expression of ANP32E significantly correlated with cell cycle‐related genes and G1/S phase‐related signatures in TNBC (cell cycle‐related gene, *P *<* *0.001; G1/S phase‐related genes, *P *<* *0.001; respectively; Fig. 4A). Moreover, ANP32E positively correlated with Ki‐67 based on the TCGA database (*r* = 0.506; *P *<* *0.001; Fig. 4B). These results indicated that ANP32E might promote proliferation in TNBC cells.

We then constructed stable MDA‐MB‐361, 4T1, and SUM159PT cell lines overexpressing or underexpressing ANP32E (Fig. S1). Using the colony formation assay, we observed that cell proliferation capacity was enhanced by overexpressing ANP32E, but suppressed by inhibiting ANP32E (Fig. 4C,D). These results suggested that ANP32E overexpression induced proliferation in TNBC cells.

As ANP32E expression significantly correlated with cell cycle‐related gene signatures, we assumed that the underlying mechanism of proliferation may be associated with the regulation of cell cycle in TNBC cells. We observed that the number of cells decreased at the peak of S phase and increased at the G0/G1 peak in ANP32E‐inhibited TNBC cells using flow cytometry (Fig. 4E). Meanwhile, the number of ANP32E‐overexpressing cells increased at the peak of S phase, but decreased at the G0/G1 peak (Fig. 4F). ANP32E overexpression improved the G1/S phase transition in TNBC cells, while ANP32E inhibition suppressed the G1/S phase transition. To further prove the oncogenic role of ANP32E, we overexpressed ANP32E in MDA‐MB‐361 which is barely expressing ANP32E and observed that ANP32E overexpression significantly enhanced the colony formation ability in the MDA‐MB‐361 cells (Fig. S2B). However, inhibiting ANP32E did not suppress MDA‐MB‐361 cell proliferation significantly (Fig. S2A). Moreover, the flow cytometry analysis also showed that ANP32E overexpression significantly improved G1/S transition in the MDA‐MB‐361 cells, while ANP32E inhibition only suppressed G1/S transition slightly (Fig. S2C,D). Cumulatively, these results suggested that ANP32E induces cell proliferation by promoting the G1/S transition in TNBC cells.

### 3.4 ANP32E downregulation suppresses tumorigenesis and proliferation in TNBC

**Figure 5:**
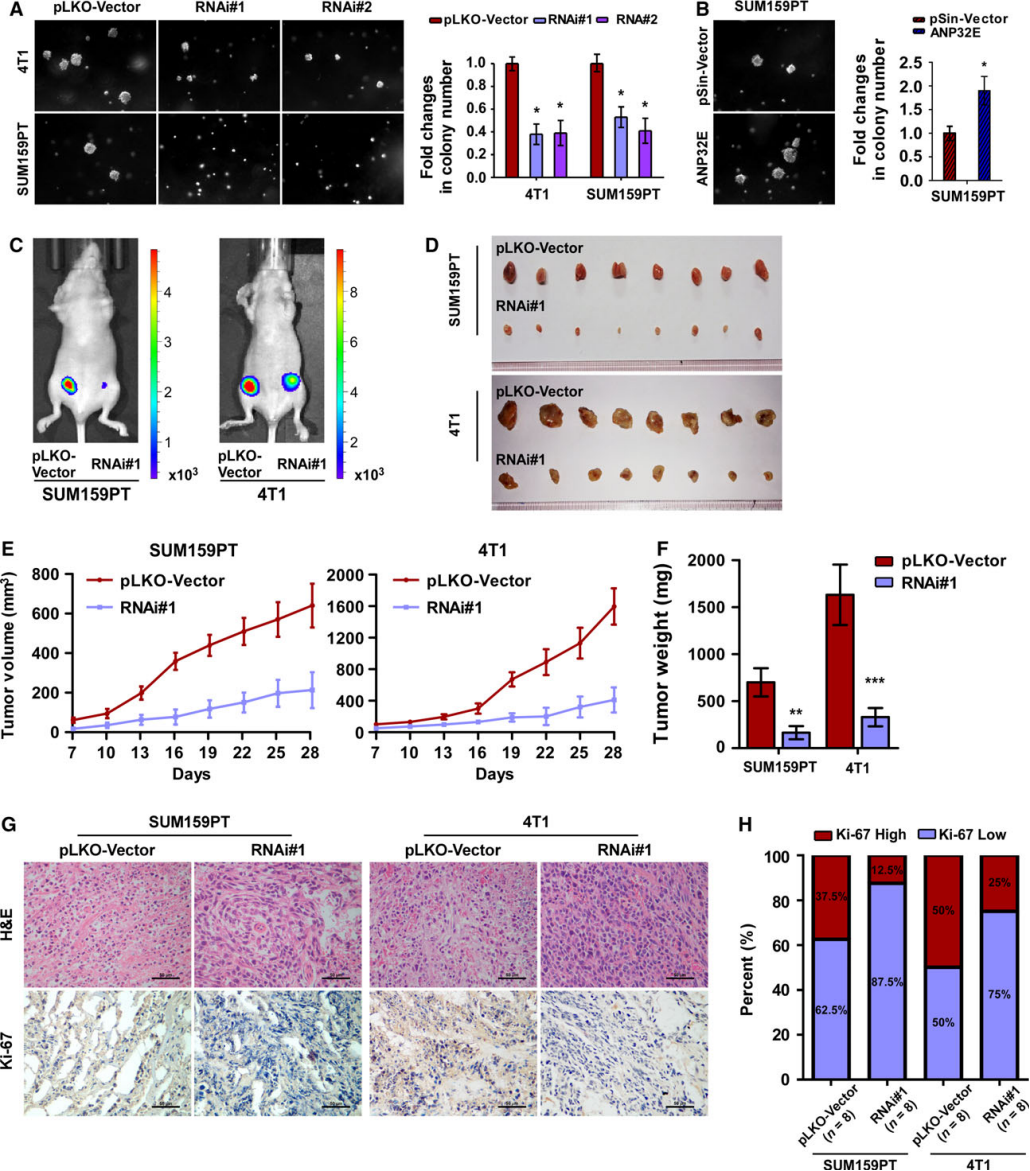
ANP32E downregulation suppresses tumorigenesis and proliferation of TNBC cells. (A, B) Representative images (left) and quantification (right) of anchorage‐independent growth colony formation for the indicated cell lines. (C) Xenograft model in nude mice. Representative images of tumor‐bearing mice (C) and tumors (D) formed by indicated cell lines. (E) Volumes of tumors formed by the indicated cells were measured every 3 days beginning at Day 7. Data were recorded as the means ± SD of three independent tests. (F) Weight of tumors formed by indicated cells were recorded as the means ± SD. (G) H&E staining and IHC staining for of Ki‐67 were performed on tumors of nude mice. (H) Quantification of Ki‐67 staining in tumors formed by SUM159PT and 4T1. *P <* *0.05, **P <* *0.01, ***P <* *0.001.

Because ANP32E influenced tumor progression *in vitro*, we next investigated the effect of ANP32E expression on the tumorigenesis of TNBC cells. Using the soft‐agar colony formation assay, we found that anchorage‐independent growth was enhanced in the ANP32E‐overexpressing cell line, but suppressed in ANP32E‐inhibited cell lines (Fig. 5A,B). Next, we subcutaneously injected SUM‐159PT/4T1 cells into mice and observed that tumors formed by ANP32E‐inhibited SUM‐159PT/4T1 cells grew much slower and were smaller than tumors formed by the negative controls (Fig. 5C–F). We assayed the expression of Ki‐67, and found that ANP32E‐inhibited tumors displayed lower expression of Ki‐67 comparing to the tumors formed by the control cell lines (Fig. 5G,H). All these implied that ANP32E inhibition suppressed tumor proliferation in TNBC.

### 3.5 ANP32E promotes the G1/S transition by upregulating E2F1

**Figure 6:**
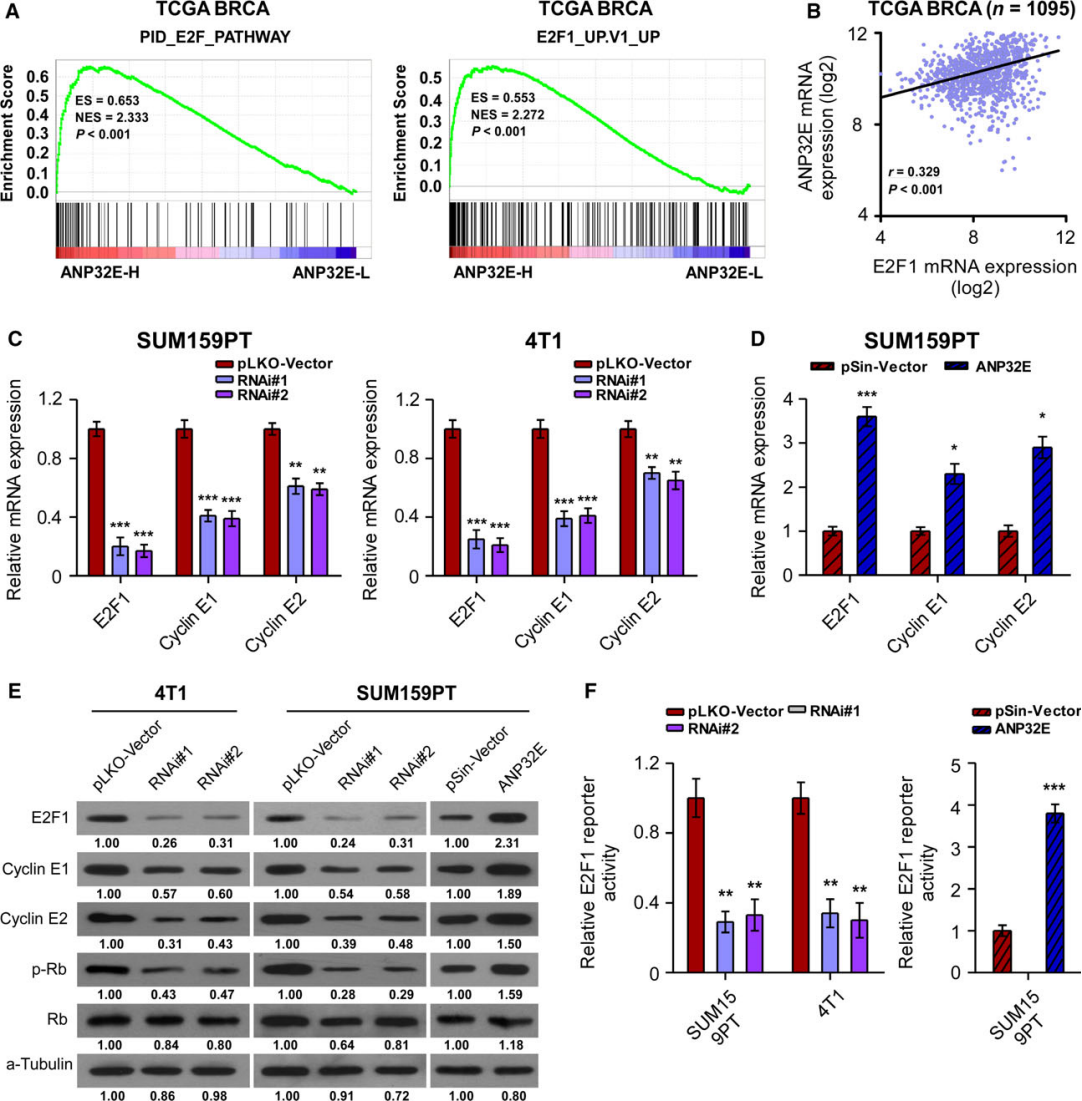
ANP32E promotes the G1/S transition by activating E2F1 expression. (A) GSEA plot showing that high ANP32E expression positively correlated with the E2F pathway‐related (PID_E2F_PATHWAY) and E2F1‐related (E2F1_UP.V1_UP) signatures based on the TCGA *BRCA* (TNBC) mRNA dataset. (B) Correlation between mRNA levels of *ANP32E* and *E2F1* based on the TCGA *BRCA* mRNA dataset. *r*, Pearson correlation coefficient; *P *<* *0.001. (C, D) The mRNA expression levels of E2F1, cyclin E1, and cyclin E2 in ANP32E‐inhibited (C) and ANP32E‐overexpressing (D) cell lines. Data were recorded as the means ± SD of three independent experiments. (E) The protein expression levels of E2F1, cyclin E1, cyclin E2, phosphorylation‐Rb (p‐Rb), and Rb in the indicated breast cancer cell lines. (F) Luciferase activity assays in SUM159PT and 4T1 cells showed that promoters of *E2F1* were repressed by the inhibition of ANP32E and activated by overexpression of ANP32E. Fold changes of the protein level were evaluated by ImageJ software (https://imagej.nih.gov/ij/). Data were obtained from three independent experiments. *P <* *0.05, **P <* *0.01, ***P <* *0.001.

To further study the molecular mechanisms of ANP32E in tumor progression, we performed a GSEA, which showed that high levels of ANP32E significantly correlated with E2F1‐related gene signatures (Fig. 6A). Furthermore, using the TCGA database, we observed that the expression of ANP32E positively correlated with the expression of E2F1 (*r* = 0.329, *P *<* *0.001; Fig. 6B). Real‐time PCR revealed that the expression of E2F1 was significantly higher in ANP32E‐overexpressing cells, but it was decreased in ANP32E‐inhibited cells (Fig. 6C,D). Consistently, western blot assays showed that the expression of ANP32E positively correlated with the protein expression levels of E2F1 (Fig. 6E). Moreover, the protein expression levels of phosphorylated Rb, which indicates the transcription activity of E2F1, increased in ANP32E‐overexpressing cells and decreased in ANP32E‐inhibited cells (Fig. 6E). Consistently, the luciferase activity reporting system showed that the promoter of E2F1 was activated by ANP32E in TNBC cells (Fig. 6F).

As ANP32E upregulated E2F1 in TNBC cells, we further explored the mechanisms underlying the promotion of the G1/S transition by ANP32E. As expected, qRT–PCR and western blot assays showed that the expression of cyclin E1 and cyclin E2, which are downstream targets of E2F1, positively correlated with ANP32E expression in TNBC cells (Fig. 6C–E). Collectively, these results suggested that ANP32E promoted the G1/S transition by upregulating E2F1 expression.

### 3.6 ANP32E promotes TNBC cell growth by upregulating E2F1 and cyclin E1/E2

**Figure 7:**
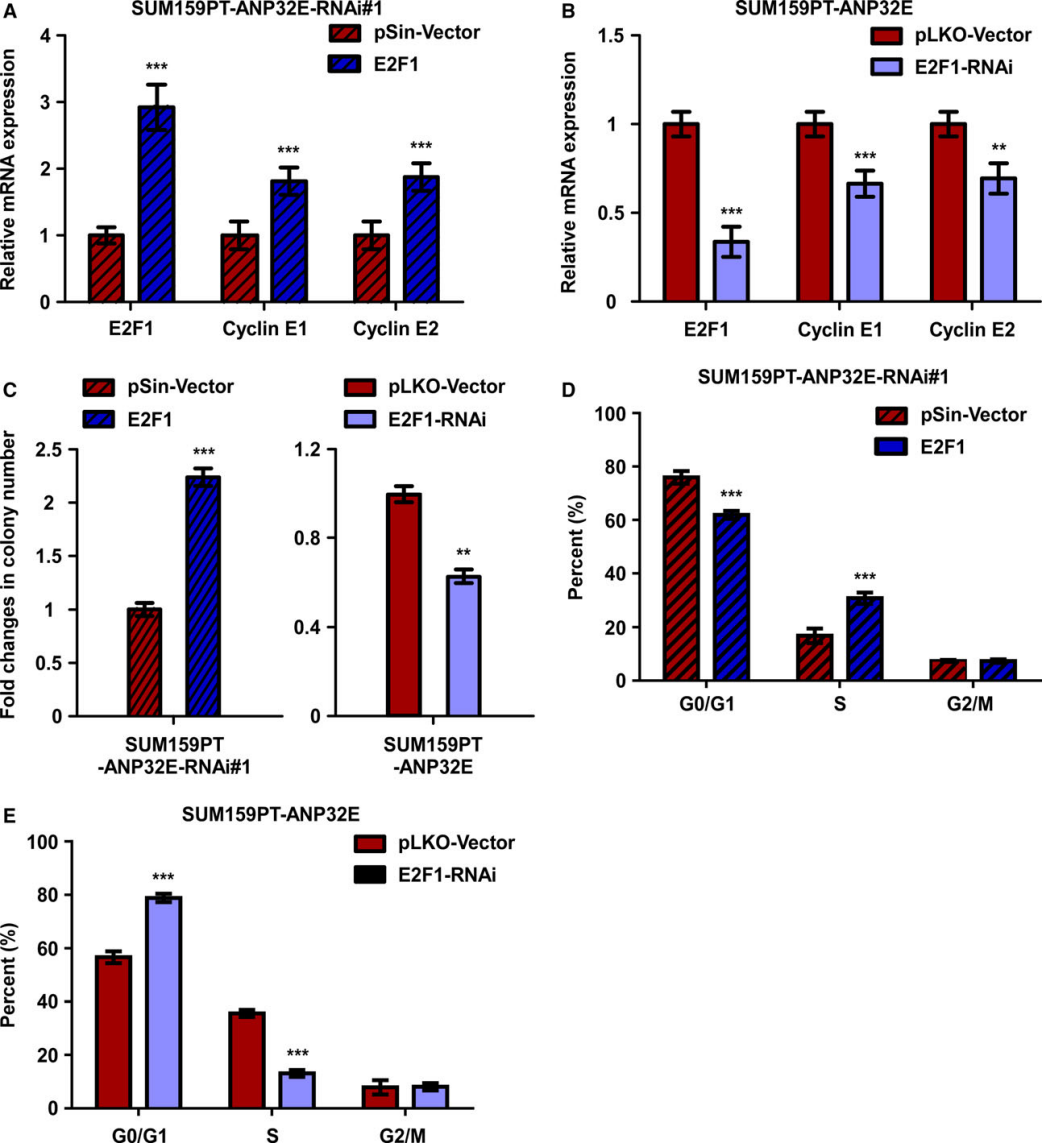
ANP32E promotes TNBC cell growth by upregulating E2F1 and cyclin E1/E2. (A, B) The mRNA expression levels of E2F1, cyclin E1, and cyclin E2 in (A) E2F1‐overexpressing and (B) E2F1‐inhibited cell lines. Data were recorded as the means ± SD of three independent experiments. (C) Quantification of colony formation (left) E2F1‐overexpressing and (right) E2F1‐inhibited cell lines. (D and E) Flow cytometric analysis of indicated cell lines. **P <* *0.01, ***P <* *0.001.

To further investigate the mechanism underlying the promotion of tumor growth by ANP32E, we restored E2F1 in ANP32E‐inhibited cells (SUM159PT and BT‐549) and inhibited E2F1 in ANP32E‐overexpressing human breast cancer cells (SUM159PT and MDA‐MB‐361) to verify the mechanistic linkage between ANP32E, E2F1, and cyclin E (CCNE) in mediating cell proliferation. As expected, E2F1 overexpression upregulated cyclin E1 and cyclin E2, while E2F1 inhibition downregulated them. (Fig. 7A,B). Colony formation and flow cytometric analysis indicated that E2F1 overexpression enhanced the growth ability of TNBC cells (SUM159PT) and that E2F1 inhibition suppressed it (Figure 7C–E). Similarly, E2F1 inhibition or E2F1 overexpression could offset the effects of ANP32E overexpression or ANP32E inhibition, respectively, on MDA‐MB‐361 and BT‐549 cell growth (Fig. S3). These results consistently showed that E2F1 is necessary for the effect of ANP32E on cancer cell growth.

## 4 Discussion

Our study has shown that ANP32E is highly expressed in TNBC cells. This correlates with poor patient outcomes and enhanced tumorigenesis as a result of upregulation of E2F1 and the induction of cell cycle progression.

Triple‐negative breast cancers display highly invasive and proliferative properties, and patients have a much higher risk for early recurrence and metastasis than others. However, as these tumors are negative for the ER, PR, and HER2 receptor, women with TNBCs were not responsive to targeted treatments (endocrine treatment or anti‐HER2 treatment) and prognostic factors predicting risk of cancer recurrence and distant metastasis in TNBC are lacking (Denkert *et al*., 2016; Jiang *et al*., 2016). Thus, searching for effective markers or potential therapeutic targets is necessary for improvement of cancer treatment in TNBC. Interestingly, our study discovered that ANP32E promotes tumorigenesis and correlates with shorter survival times and higher risks of disease relapse in TNBC. This is consistent with a previous report that the expression of ANP32E is associated with lung metastasis in breast cancer. Statistical analyses showed that ANP32E expression is an independent prognostic factor for TNBC as opposed to non‐TNBC. Moreover, we observed that the expression of ANP32E is significantly higher in TNBC tissues compared to other breast cancer tissues. All of these data suggest that ANP32E could be an effective prognostic factor for patients with TNBC.

ANP32E is involved in many physiological processes, such as cell proliferation and apoptosis in mammalian cells (Li *et al*., 2017; Obri *et al*., 2014). Although the significance of ANP32E in breast cancer is poorly studied, several articles have reported an important role for ANP32E in myeloma and gastric cancer. We observed that ANP32E correlated with cell cycle‐related gene signature. ANP32E overexpression induces G1/S phase transition and promoted tumor proliferation in TNBC cells. Moreover, the tumor‐promoting effect of ANP32E was weakened by inhibiting ANP32E expression. As capacity of proliferation is a critical hallmark for tumor progression, ANP32E could be a key factor in the process of tumor dissemination and colonization. These results suggested that ANP32E promotes tumor progression by inducing cell cycle progression, which explained the phenomenon that high expression of ANP32E was significantly correlated with the high risk of cancer recurrence and metastasis in TNBC. Further studies are required to study the potential role of ANP32E in cancer metastasis.

As previously reported, ANP32E can act as a histone chaperone to remove H2A.Z, a histone variant, from nucleosomes can cause transcriptional activation of gene (Farris *et al*., 2005; Obri *et al*., 2014). Consistently, our study showed that ANP32E overexpression was significantly correlated with transcriptional activation of E2F1 in TNBC cells. E2F1 is a transcription factor that regulates cyclins (cyclin E1/2 and cyclin A2). Cyclins (such as cyclin E1/2 and cyclin A2) are cell cycle regulators that form a complex with CDK to induce cell cycle progression (Bloom and Cross, 2007; Hochegger *et al*., 2008; Malumbres and Barbacid, 2009). CDK–cyclin complexes could reversely activate E2F transcription factors by phosphorylating and inactivating Rb, which promotes a positive feedback (Bjorklund *et al*., 2006; Leone *et al*., 1997; Zielke *et al*., 2011). In our study, expression of cyclin E1/2 was positively correlated with ANP32E expression. All these data indicate that ANP32E induces cell cycle progression by upregulating E2F1, which subsequently promotes tumor proliferation in TNBC. Otherwise, Anp32e promoted breast cancer cell proliferation and anti‐apoptosis ability by inhibiting phosphatase 2A, a tumor suppressor that represses the PI3K/AKT pathway, in a mouse model which indicated that ANP32E might promote cell cycle progression through multiple pathways (Nakahata *et al*., 2014; Switzer *et al*., 2011). All in all, our study has firstly reported the mechanism that ANP32E promotes cell cycle progression and tumorigenesis by transcriptionally activating E2F1.

At present, the lack of effective prognostic predictors and therapeutic targets remains the major challenge to TNBC therapy. Interestingly, we discovered that ANP32E is a negative prognostic factor, where high ANP32E expression was correlated with high risk of cancer recurrence and distant metastasis. We were able to identify patients with high mortality risk by detecting ANP32E and arranged more aggressive treatments and frequent disease surveillance for these patients. Further, we observed that ANP32E is a critical factor in tumor progression in that it induces tumorigenesis by promoting cell cycle progression, which explains why ANP32E expression predicts poor outcomes in TNBC. These results indicate that inhibiting ANP32E could be a potential therapeutic method in TNBC, and finding an effective method of inhibiting ANP32E expression would be of great value. Recently, CRISPR–Cas9, a genome editing system that can recognize and edit gene sequences, had been widely studied in animal models and cancer cell lines to knock out gene expression (Shalem *et al*., 2014). Moreover, the first clinical trial using CRISPR to edit out genes has been launched for cancer treatment (Cyranoski, 2016). Therefore, CRISPR–Cas9 could be an effective tool for inhibiting ANP32E in TNBC.

## 5 Conclusions

In conclusion, this is the first study to show that ANP32E is a negative prognostic factor that enhances tumorigenesis and cancer proliferation by promoting the G1/S transition in TNBC. TNBC patients with high expression of ANP32E might have a higher risk of disease recurrence and distant metastasis. Thus, they may consider undergoing more aggressive treatments and frequent disease surveillance. More studies of the molecular mechanisms of ANP32E in tumor progression and metastasis may be beneficial to explore novel therapeutic targets in TNBC.

## Ethics approval and consent to participate

Ethical approval was obtained from the respective institutional review boards of the Ethics Committee of Sun Yat‐sen University Cancer Center. All patients provided written informed consent to participate in this study.

## Consent for publication

Not applicable.

## Availability of data and materials

The datasets supporting the conclusions of this article were deposited in the Research Data Deposit system of Sun Yat‐sen University Cancer and can be obtained from the corresponding authors on reasonable request.

## Author contributions

ZYY and XW conceived the idea; ZCX, XW, and LBS conceived and designed the experiments; ZCX and LPY wrote the manuscript; ZCX, LPY, FYL, GZD, WS, and XQW performed the experiments and analyzed the data; CYL, and GZD helped in interpreting the results and writing the manuscript; ZYH and YHX revised the manuscript based on the comments of reviewers. All authors approved the final version.

## Supporting information


**Fig. S1**. ANP32E protein expression in TNBC cell lines.

**Fig. S2**. ANP32E promotes cell proliferation in breast cancer cells.

**Fig. S3.** ANP32E promotes TNBC cell growth by upregulating E2F1 and cyclin E1/E2.

**Table S1**. Correlation between ANP32E expression and clinicopathologic characteristics of breast cancer patient.
**Table S2**. HR for women with Non‐TNBC (univariate and multivariate).
**Table S3**. Subgroup survival analysis of ANP32E expression in breast cancer (univariate analysis).
**Table S4**. Real‐time PCR primers in our study.

## References

[mol212202-bib-0001] Badwe R , Hawaldar R , Nair N , Kaushik R , Parmar V , Siddique S , Budrukkar A , Mittra I and Gupta S (2015) Locoregional treatment versus no treatment of the primary tumour in metastatic breast cancer: an open‐label randomised controlled trial. Lancet Oncol 16, 1380–1388. 26363985 10.1016/S1470-2045(15)00135-7

[mol212202-bib-0002] Bi H , Li S , Qu X , Wang M , Bai X , Xu Z , Ao X , Jia Z , Jiang X , Yang Y *et al* (2015) DEC1 regulates breast cancer cell proliferation by stabilizing cyclin E protein and delays the progression of cell cycle S phase. Cell Death Dis 6, e1891. 26402517 10.1038/cddis.2015.247 PMC4650443

[mol212202-bib-0003] Bjorklund M , Taipale M , Varjosalo M , Saharinen J , Lahdenpera J and Taipale J (2006) Identification of pathways regulating cell size and cell‐cycle progression by RNAi. Nature 439, 1009–1013. 16496002 10.1038/nature04469

[mol212202-bib-0004] Bloom J and Cross FR (2007) Multiple levels of cyclin specificity in cell‐cycle control. Nat Rev Mol Cell Biol 8, 149–160. 17245415 10.1038/nrm2105

[mol212202-bib-0005] Cam H and Dynlacht BD (2003) Emerging roles for E2F: beyond the G1/S transition and DNA replication. Cancer Cell 3, 311–316. 12726857 10.1016/s1535-6108(03)00080-1

[mol212202-bib-0006] Chan E and Nimnual AS (2010) Deregulation of the cell cycle by breast tumor kinase (Brk). Int J Cancer 127, 2723–2731. 20162673 10.1002/ijc.25263

[mol212202-bib-0007] Cheang MC , Chia SK , Voduc D , Gao D , Leung S , Snider J , Watson M , Davies S , Bernard PS , Parker JS *et al* (2009) Ki67 index, HER2 status, and prognosis of patients with luminal B breast cancer. J Natl Cancer Inst 101, 736–750. 19436038 10.1093/jnci/djp082 PMC2684553

[mol212202-bib-0008] Cianfrocca M and Gradishar W (2009) New molecular classifications of breast cancer. CA Cancer J Clin 59, 303–313. 19729680 10.3322/caac.20029

[mol212202-bib-0009] Cyranoski D (2016) CRISPR gene‐editing tested in a person for the first time. Nature 539, 479. 27882996 10.1038/nature.2016.20988

[mol212202-bib-0010] Denkert C , Liedtke C , Tutt A , von Minckwitz G (2016) Molecular alterations in triple‐negative breast cancer—the road to new treatment strategies. Lancet 389, 2430–2442. 27939063 10.1016/S0140-6736(16)32454-0

[mol212202-bib-0011] Dietze EC , Sistrunk C , Miranda‐Carboni G , O'Regan R and Seewaldt VL (2015) Triple‐negative breast cancer in African‐American women: disparities versus biology. Nat Rev Cancer 15, 248–254. 25673085 10.1038/nrc3896 PMC5470637

[mol212202-bib-0012] Duan Y , Tian L , Gao Q , Liang L , Zhang W , Yang Y , Zheng Y , Pan E , Li S and Tang N (2016) Chromatin remodeling gene ARID2 targets cyclin D1 and cyclin E1 to suppress hepatoma cell progression. Oncotarget 7, 45863–45875. 27351279 10.18632/oncotarget.10244 PMC5216766

[mol212202-bib-0013] Farris SD , Rubio ED , Moon JJ , Gombert WM , Nelson BH and Krumm A (2005) Transcription‐induced chromatin remodeling at the c‐myc gene involves the local exchange of histone H2A.Z. J Biol Chem 280, 25298–25303. 15878876 10.1074/jbc.M501784200

[mol212202-bib-0014] Foulkes WD , Smith IE and Reis‐Filho JS (2010) Triple‐negative breast cancer. N Engl J Med 363, 1938–1948. 21067385 10.1056/NEJMra1001389

[mol212202-bib-0015] Gursoy‐Yuzugullu O , Ayrapetov MK and Price BD (2015) Histone chaperone Anp32e removes H2A.Z from DNA double‐strand breaks and promotes nucleosome reorganization and DNA repair. Proc Natl Acad Sci 112, 7507–7512. 26034280 10.1073/pnas.1504868112 PMC4475971

[mol212202-bib-0016] Hanahan D and Weinberg RA (2011) Hallmarks of cancer: the next generation. Cell 144, 646–674. 21376230 10.1016/j.cell.2011.02.013

[mol212202-bib-0017] Hochegger H , Takeda S and Hunt T (2008) Cyclin‐dependent kinases and cell‐cycle transitions: does one fit all? Nat Rev Mol Cell Biol 9, 910–916. 18813291 10.1038/nrm2510

[mol212202-bib-0018] Hunt KK , Karakas C , Ha MJ , Biernacka A , Yi M , Sahin AA , Adjapong O , Hortobagyi GN , Bondy ML , Thompson PA *et al* (2016) Cytoplasmic cyclin E predicts recurrence in patients with breast cancer. Clin Cancer Res 23, 2991–3002. 27881578 10.1158/1078-0432.CCR-16-2217 PMC5441976

[mol212202-bib-0019] Jiang M , Ma Y , Ni X , Cao G , Ji C , Cheng H , Tang R , Xie Y and Mao Y (2002) Molecular cloning and characterization of a novel human gene (ANP32E alias LANPL) from human fetal brain. Cytogenet Genome Res 97, 68–71. 12438741 10.1159/000064058

[mol212202-bib-0020] Jiang T , Shi W , Wali VB , Pongor LS , Li C , Lau R , Gyorffy B , Lifton RP , Symmans WF , Pusztai L *et al* (2016) Predictors of chemosensitivity in triple negative breast cancer: an integrated genomic analysis. PLoS Med 13, e1002193. 27959926 10.1371/journal.pmed.1002193 PMC5154510

[mol212202-bib-0021] Keyomarsi K , Tucker SL , Buchholz TA , Callister M , Ding Y , Hortobagyi GN , Bedrosian I , Knickerbocker C , Toyofuku W , Lowe M *et al* (2002) Cyclin E and survival in patients with breast cancer. N Engl J Med 347, 1566–1575. 12432043 10.1056/NEJMoa021153

[mol212202-bib-0022] Kobe B and Kajava AV (2001) The leucine‐rich repeat as a protein recognition motif. Curr Opin Struct Biol 11, 725–732. 11751054 10.1016/s0959-440x(01)00266-4

[mol212202-bib-0023] Landemaine T , Jackson A , Bellahcene A , Rucci N , Sin S , Abad BM , Sierra A , Boudinet A , Guinebretiere JM , Ricevuto E *et al* (2008) A six‐gene signature predicting breast cancer lung metastasis. Cancer Res 68, 6092–6099. 18676831 10.1158/0008-5472.CAN-08-0436

[mol212202-bib-0024] Leone G , DeGregori J , Sears R , Jakoi L and Nevins JR (1997) Myc and Ras collaborate in inducing accumulation of active cyclin E/Cdk2 and E2F. Nature 387, 422–426. 9163430 10.1038/387422a0

[mol212202-bib-0025] Li M , Ma F , Wang J , Li Q , Zhang P , Yuan P , Luo Y , Cai R , Fan Y , Chen S *et al* (2018) Genetic polymorphisms of autophagy‐related gene 5 (ATG5) rs473543 predict different disease‐free survivals of triple‐negative breast cancer patients receiving anthracycline‐ and/or taxane‐based adjuvant chemotherapy. Chin J Cancer 37, 4. 29382381 10.1186/s40880-018-0268-1 PMC5791378

[mol212202-bib-0026] Li P , Xu T , Zhou X , Liao L , Pang G , Luo W , Han L , Zhang J , Luo X , Xie X *et al* (2017) Downregulation of miRNA‐141 in breast cancer cells is associated with cell migration and invasion: involvement of ANP32E targeting. Cancer Med 6, 662–672. 28220627 10.1002/cam4.1024 PMC5345683

[mol212202-bib-0027] Li J , Zhang N , Song LB , Liao WT , Jiang LL , Gong LY , Wu J , Yuan J , Zhang HZ , Zeng MS *et al* (2008) Astrocyte elevated gene‐1 is a novel prognostic marker for breast cancer progression and overall patient survival. Clin Cancer Res 14, 3319–3326. 18519759 10.1158/1078-0432.CCR-07-4054

[mol212202-bib-0028] Lin H , Dai T , Xiong H , Zhao X , Chen X , Yu C , Li J , Wang X and Song L (2010) Unregulated miR‐96 induces cell proliferation in human breast cancer by downregulating transcriptional factor FOXO3a. PLoS ONE 5, e15797. 21203424 10.1371/journal.pone.0015797 PMC3009749

[mol212202-bib-0029] Lin C , Song L , Liu A , Gong H , Lin X , Wu J , Li M and Li J (2015) Overexpression of AKIP1 promotes angiogenesis and lymphangiogenesis in human esophageal squamous cell carcinoma. Oncogene 34, 384–393. 24413079 10.1038/onc.2013.559

[mol212202-bib-0030] Lin C , Wu Z , Lin X , Yu C , Shi T , Zeng Y , Wang X , Li J and Song L (2011) Knockdown of FLOT1 impairs cell proliferation and tumorigenicity in breast cancer through upregulation of FOXO3a. Clin Cancer Res 17, 3089–3099. 21447726 10.1158/1078-0432.CCR-10-3068

[mol212202-bib-0031] Magbanua MJ , Wolf DM , Yau C , Davis SE , Crothers J , Au A , Haqq CM , Livasy C , Rugo HS , Investigators IST *et al* (2015) Serial expression analysis of breast tumors during neoadjuvant chemotherapy reveals changes in cell cycle and immune pathways associated with recurrence and response. Breast Cancer Res 17, 73. 26021444 10.1186/s13058-015-0582-3 PMC4479083

[mol212202-bib-0032] Malumbres M and Barbacid M (2009) Cell cycle, CDKs and cancer: a changing paradigm. Nat Rev Cancer 9, 153–166. 19238148 10.1038/nrc2602

[mol212202-bib-0033] Mao Z , Pan L , Wang W , Sun J , Shan S , Dong Q , Liang X , Dai L , Ding X , Chen S *et al* (2014) Anp32e, a higher eukaryotic histone chaperone directs preferential recognition for H2A.Z. Cell Res 24, 389–399. 24613878 10.1038/cr.2014.30 PMC3975505

[mol212202-bib-0034] Matilla A , Radrizzani M (2005) The Anp32 family of proteins containing leucine‐rich repeats. Cerebellum (London, England) 4, 7–18. 10.1080/14734220410019020 15895553

[mol212202-bib-0035] Muellner MK , Mair B , Ibrahim Y , Kerzendorfer C , Lechtermann H , Trefzer C , Klepsch F , Muller AC , Leitner E , Macho‐Maschler S *et al* (2015) Targeting a cell state common to triple‐negative breast cancers. Mol Syst Biol 11, 789. 25699542 10.15252/msb.20145664 PMC4358660

[mol212202-bib-0036] Nakahata S , Ichikawa T , Maneesaay P , Saito Y , Nagai K , Tamura T , Manachai N , Yamakawa N , Hamasaki M , Kitabayashi I *et al* (2014) Loss of NDRG2 expression activates PI3K‐AKT signalling via PTEN phosphorylation in ATLL and other cancers. Nat Commun 5, 3393. 24569712 10.1038/ncomms4393 PMC3948061

[mol212202-bib-0037] Obri A , Ouararhni K , Papin C , Diebold ML , Padmanabhan K , Marek M , Stoll I , Roy L , Reilly PT , Mak TW *et al* (2014) ANP32E is a histone chaperone that removes H2A.Z from chromatin. Nature 505, 648–653. 24463511 10.1038/nature12922

[mol212202-bib-0038] Ossovskaya V , Wang Y , Budoff A , Xu Q , Lituev A , Potapova O , Vansant G , Monforte J and Daraselia N (2011) Exploring molecular pathways of triple‐negative breast cancer. Genes Cancer 2, 870–879. 22593799 10.1177/1947601911432496 PMC3352156

[mol212202-bib-0039] Otto T and Sicinski P (2017) Cell cycle proteins as promising targets in cancer therapy. Nat Rev Cancer 17, 93–115. 28127048 10.1038/nrc.2016.138 PMC5345933

[mol212202-bib-0040] Park YH , Lee SJ , Cho EY , Choi YL , Lee JE , Nam SJ , Yang JH , Shin JH , Ko EY , Han BK *et al* (2011) Clinical relevance of TNM staging system according to breast cancer subtypes. Ann Oncol 22, 1554–1560. 21242587 10.1093/annonc/mdq617

[mol212202-bib-0041] Qiu J , Xue X , Hu C , Xu H , Kou D , Li R and Li M (2016) Comparison of clinicopathological features and prognosis in triple‐negative and non‐triple negative breast cancer. J Cancer 7, 167–173. 26819640 10.7150/jca.10944 PMC4716849

[mol212202-bib-0042] Radrizzani M , Vila‐Ortiz G , Cafferata EG , Di Tella MC , Gonzalez‐Guerrico A , Perandones C , Pivetta OH , Carminatti H , Idoyaga Vargas VP and Santa‐Coloma TA (2001) Differential expression of CPD1 during postnatal development in the mouse cerebellum. Brain Res 907, 162–174. 11430900 10.1016/s0006-8993(01)02351-4

[mol212202-bib-0043] Shalem O , Sanjana NE , Hartenian E , Shi X , Scott DA , Mikkelson T , Heckl D , Ebert BL , Root DE , Doench JG *et al* (2014) Genome‐scale CRISPR‐Cas9 knockout screening in human cells. Science 343, 84–87. 24336571 10.1126/science.1247005 PMC4089965

[mol212202-bib-0044] Stoeck A , Lejnine S , Truong A , Pan L , Wang H , Zang C , Yuan J , Ware C , MacLean J , Garrett‐Engele PW *et al* (2014) Discovery of biomarkers predictive of GSI response in triple‐negative breast cancer and adenoid cystic carcinoma. Cancer Discov 4, 1154–1167. 25104330 10.1158/2159-8290.CD-13-0830 PMC4184927

[mol212202-bib-0045] Switzer CH , Glynn SA , Ridnour LA , Cheng RY , Vitek MP , Ambs S and Wink DA (2011) Nitric oxide and protein phosphatase 2A provide novel therapeutic opportunities in ER‐negative breast cancer. Trends Pharmacol Sci 32, 644–651. 21893353 10.1016/j.tips.2011.07.001 PMC3380363

[mol212202-bib-0046] Taylor‐Harding B , Aspuria PJ , Agadjanian H , Cheon DJ , Mizuno T , Greenberg D , Allen JR , Spurka L , Funari V , Spiteri E *et al* (2015) Cyclin E1 and RTK/RAS signaling drive CDK inhibitor resistance via activation of E2F and ETS. Oncotarget 6, 696–714. 25557169 10.18632/oncotarget.2673 PMC4359249

[mol212202-bib-0047] Vuaroqueaux V , Urban P , Labuhn M , Delorenzi M , Wirapati P , Benz CC , Flury R , Dieterich H , Spyratos F , Eppenberger U *et al* (2007) Low E2F1 transcript levels are a strong determinant of favorable breast cancer outcome. Breast Cancer Res 9, R33. 17535433 10.1186/bcr1681 PMC1929097

[mol212202-bib-0048] de Wit J , Hong W , Luo L and Ghosh A (2011) Role of leucine‐rich repeat proteins in the development and function of neural circuits. Annu Rev Cell Dev Biol 27, 697–729. 21740233 10.1146/annurev-cellbio-092910-154111

[mol212202-bib-0049] Ye L , Guo L , He Z , Wang X , Lin C , Zhang X , Wu S , Bao Y , Yang Q , Song L *et al* (2016) Upregulation of E2F8 promotes cell proliferation and tumorigenicity in breast cancer by modulating G1/S phase transition. Oncotarget 7, 23757–23771. 26992224 10.18632/oncotarget.8121 PMC5029661

[mol212202-bib-0050] Zielke N , Kim KJ , Tran V , Shibutani ST , Bravo MJ , Nagarajan S , van Straaten M , Woods B , von Dassow G , Rottig C *et al* (2011) Control of *Drosophila* endocycles by E2F and CRL4(CDT2). Nature 480, 123–127. 22037307 10.1038/nature10579 PMC3330263

