# Supplementary figures and images for: ANP32E induces tumorigenesis of triple‐negative breast cancer cells by upregulating E2F1
Source: Mol Oncol. 2018 Apr 18;12(6):896–912. doi: 10.1002/1878-0261.12202 (PMC5983205; doi:10.1002/1878-0261.12202)

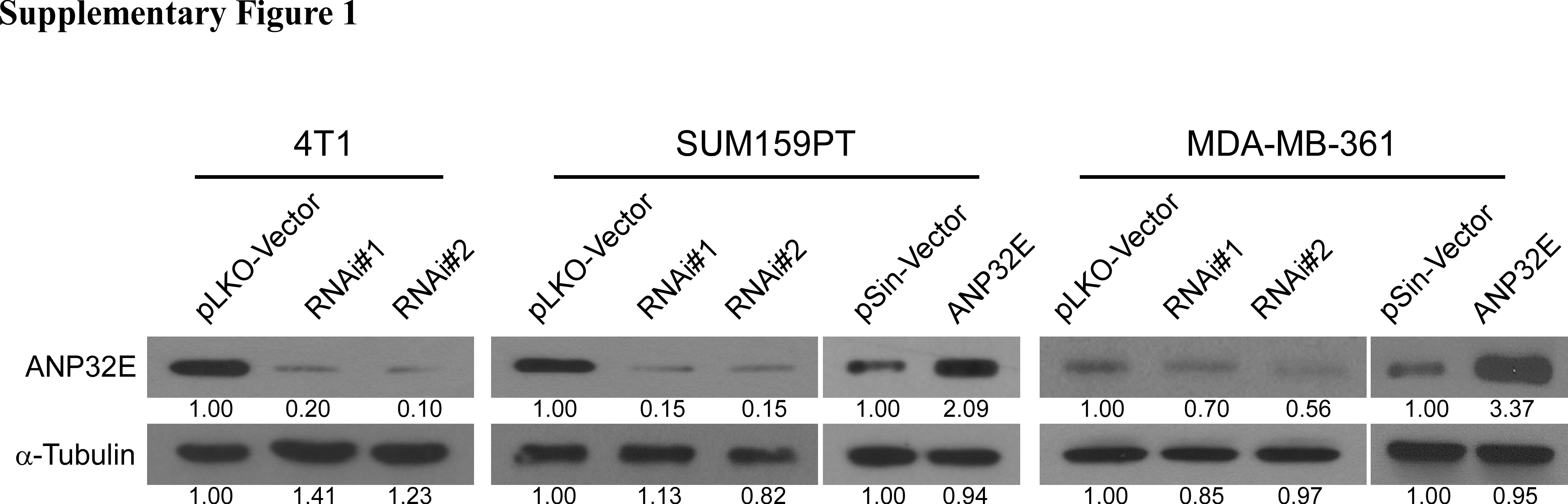

Supplement: Supplementary file 1 — Fig. S1. ANP32E protein expression in TNBC cell lines. [file MOL2-12-896-s001.tif]

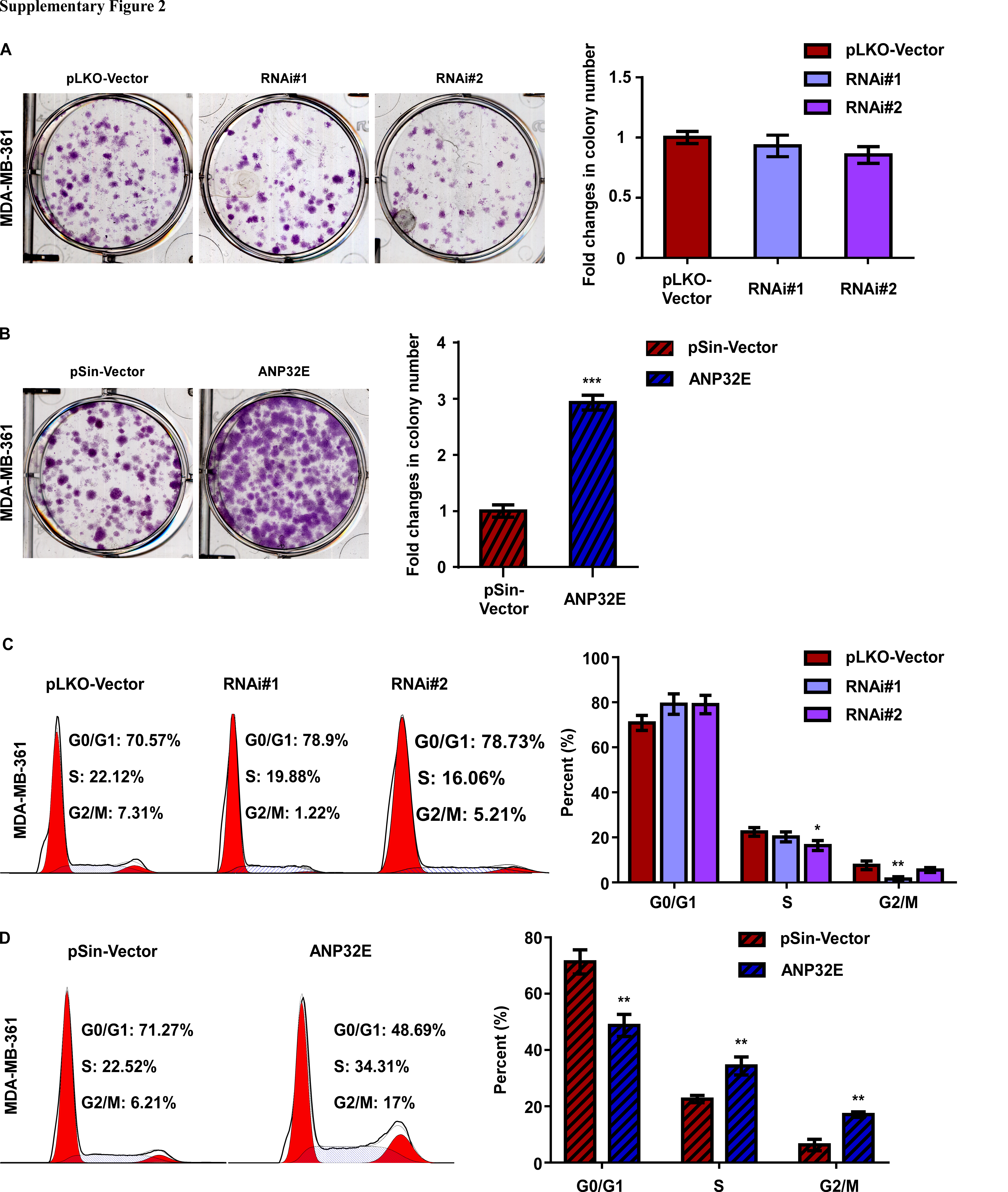

Supplement: Supplementary file 2 — Fig. S2. ANP32E promotes cell proliferation in breast cancer cells. [file MOL2-12-896-s002.tif]

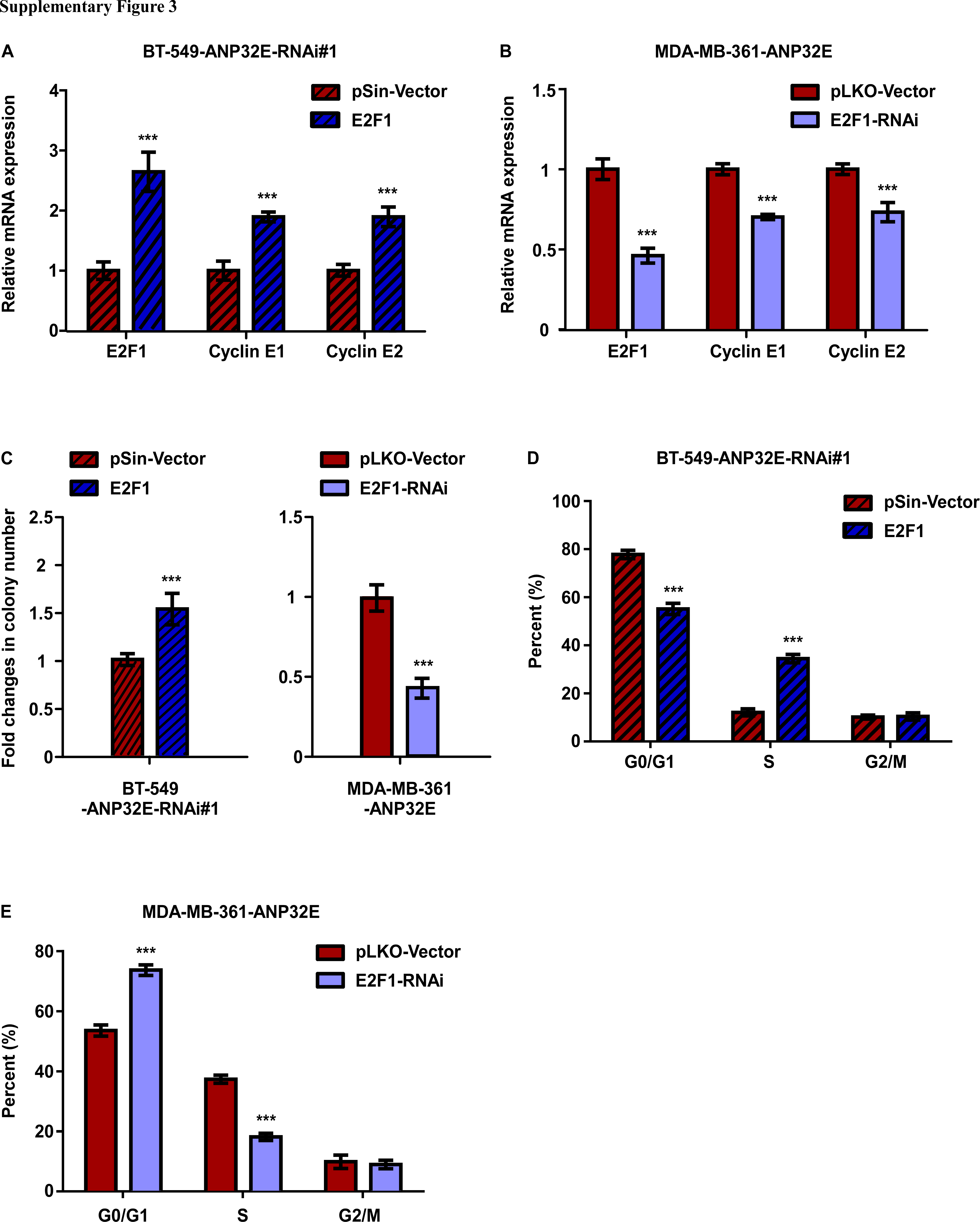

Supplement: Supplementary file 3 — Fig. S3. ANP32E promotes TNBC cell growth by upregulating E2F1 and cyclin E1/E2. [file MOL2-12-896-s003.tif]
