# Supplementary material for: ANP32E induces tumorigenesis of triple‐negative breast cancer cells by upregulating E2F1
Source: Mol Oncol. 2018 Apr 18;12(6):896–912. doi: 10.1002/1878-0261.12202 (PMC5983205; doi:10.1002/1878-0261.12202)
Supplement: Supplementary file 4 — Table S1. Correlation between ANP32E expression and clinicopathologic characteristics of breast cancer patient. Table S2. HR for women with Non‐TNBC (univariate and multivariate). Table S3. Subgroup survival analysis of ANP32E expression in breast cancer (univariate analysis). Table S4. Real‐time PCR primers in our study. [file MOL2-12-896-s004.docx]

| **Table S1. Correlation between ANP32E expression and clinicopathologic characteristics of breast cancer patient.** | | | | | | |
| --- | --- | --- | --- | --- | --- | --- |
| **Characteristics** | | **ANP32E** | | | | **(*P* value)** |
|  |  | **Low or none** | | **High** | |  |
| **Age(y)** | | | | | | 0.115 |
| ≤45 |  | 115 | | 73 | |  |
| >45 |  | 125 | | 109 | |  |
| **T classification** | | | | | | 0.297 |
| T_1-2_ |  | 216 | | 155 | |  |
| T_3-4_ |  | 18 | | 19 | |  |
| **N classification** | | | | | | 0.076 |
| N_0_ |  | 127 | | 76 | |  |
| N_1-2_ |  | 86 | | 80 | |  |
| N_3_ |  | 27 | | 26 | |  |
| **Estrogen receptor** | | | | | | ***<0.001*** |
| Positive | | 150 | | 143 | |  |
| Negative | | 90 | | 39 | |  |
| **Progesterone receptor** | | | | | | ***<0.001*** |
| Positive | | 151 | | 146 | |  |
| Negative | | 89 | | 36 | |  |
| **Her-2 receptor** | |  |  |  |  | 0.455 |
| Amplification | | 38 | | 21 | |  |
| Non-amplification | | 192 | | 152 | |  |
| 2+ | | 10 | | 9 | |  |
| **TNBC** | |  |  |  |  | ***<0.001*** |
| Yes | | 117 | | 121 | |  |
| No | | 123 | | 61 | |  |
| **Menses statuse** | | | | | | 0.023 |
| Premenopausal | | 147 | | 91 | |  |
| Menopausal | | 93 | | 91 | |  |
| **Vital status (at follow-up)** | |  |  |  |  | ***< 0.001*** |
| Alive | | 213 | | 111 | |  |
| Dead | | 27 | | 71 | |  |
| **Relapse status** | |  |  |  |  | ***< 0.001*** |
| Relapse | | 30 | | 80 | |  |
| Relapse-free | | 210 | | 102 | |  |

**Abbreviation:** TNBC = triple-negative breast cancer

| **Table S2. Hazard ratio for women with Non-TNBC (univariate and multivariate)** | | | | |
| --- | --- | --- | --- | --- |
| **Characteristic** | **Non-TNBC** | | | |
|  | **Univariate HRs (95%CI)** | ***P*** | **Multivariate HRs (95%CI)** | ***P*** |
| **Age (>45 vs ≤45)** | 1.276  (0.725-2.245) | 0.398 | NA | NA |
| **T classification (T_3-4_ vs T_1-2_)** | 1.203  (0.478-3.028) | 0.694 | NA | NA |
| **N classification** |  | ***< 0.001*** |  | ***< 0.001*** |
| N_0_ | 1 |  | 1 |  |
| N_1-2_ | 2.718  (1.293-5.716) | 0.008 | 2.458  (1.154-5.235) | 0.02 |
| N_3_ | 7.296  (3.379-15.752) | < 0.001 | 6.403  (2.928-14.006) | < 0.001 |
| **ER (+ vs -)** | 0.495  (0.285-0.861) | ***0.013*** | 0.537  (0.307-0.938) | ***0.029*** |
| **PR (+ vs -)** | 0.703  (0.401-1.231) | 0.218 | NA | NA |
| **Her-2 receptor** |  | ***0.042*** |  | 0.538 |
| No-amplification | 1 |  | 1 |  |
| Amplification | 1.911  (1.062-3.439) | 0.031 | NA | 0.934 |
| 2+ | 2.295  (0.985-5.348) | 0.054 | NA | 0.300 |
| **Menopause statuse (Menopause vs menses)** | 1.336  (0.775-2.302) | 0.297 | NA | NA |
| **Expression of ANP32E (High vs low)** | 2.993  (1.725-5.192) | ***< 0.001*** | 2.346  (1.337-4.118) | ***0.003*** |

**Abbreviation:** TNBC = triple-negative breast cancer, ER = estrogen receptor, PR = progesterone receptor, HRs = hazard ratios, CI = confidence interval

| **Table S3. Subgroup survival analysis of ANP32E expression in breast cancer (univariate analysis)** | | | |
| --- | --- | --- | --- |
| **Characteristic** | **High expression vs low expression（HRs）** | **95%CI** | ***P* Value** |
| **TNBC** | 10.349 | 4.079-26.253 | <0.001 |
| **HR: Negative** | 6.421 | 3.253-12.675 | <0.001 |
| **HER-2: Negative** | 6.186 | 3.431-11.155 | < 0.001 |
| **Age** |  |  |  |
| ≤45 | 4.130 | 1.954-8.731 | < 0.001 |
| >45 | 4.172 | 2.399-7.255 | < 0.001 |
| **Menopause status** |  |  |  |
| Premenopausal | 4.040 | 2.138-7.635 | < 0.001 |
| Menopausal | 4.013 | 2.194-7.673 | < 0.001 |

**Abbreviation:** TNBC = triple-negative breast cancer, HR = hormone receptor, HRs = hazard ratios, CI = confidence interval

**Table S4. Real-time PCR primers in our study**

| **Gene** | **Forward primer (5'-3')** | **Reverse primer (5'-3')** |
| --- | --- | --- |
| **ANP32E** | GGAGGAGGTGACAGAGTTAG | GGGCCAGCGAACTTAGTTCC |
| **E2F1** | CCGTGGACTCTTCGGAGAAC | ATCCCACCTACGGTCTCCTC |
| **Cyclin E1** | CCCCATCATGCCGAGGGAG | CCTTCCTCTTCCTGGAGCGA |
| **Cyclin E2** | TAGCTGGTCTGGCGAGGTTT | ACAGGTGGCCAACAATTCCT |
| **GAPDH** | GACTCATGACCACAGTCCATGC | AGAGGCAGGGATGATGTTCTG |
